# Automatic identification and explanation of root causes on COVID-19 index anomalies

**DOI:** 10.1016/j.mex.2022.101960

**Published:** 2022-12-08

**Authors:** F.K. Sufi

**Affiliations:** School of Public Health and Preventive Medicine, Monash University, Melbourne, VIC 3004, Australia

**Keywords:** Multidimensional analysis of COVID-19, Anomaly detection on COVID-19 posts, Deep learning on COVID-19 dimensions, Evidence based decision making, Decision support system, Anomaly detection on COVID-19 indexes

## Abstract

This paper reports a method for automatically identifying, analyzing and explaining anomalies in different indexes of COVID-19 crisis using Artificial Intelligence (AI) based techniques. With systematic application of News sensor, language detection & translation, Keyword-based extraction of COVID-19 indexes, Convolutional Neural Network (CNN) based anomaly detection, and Natural Language Processing (NLP) based explanation methods, this paper demonstrates a methodological solution for strategic decision makers to make evidence-based policy decisions on COVID-19 (in multiple dimensions like Travel, Vaccine, Medical etc.). Firstly, COVID-19 related News is fetched from multiple sources in multiple languages. Then, AI-based language detection and translation process automatically translates these News and posts in real-time. Next, COVID-19 related News and posts are segregated in multiple groups using pre-defined keywords for creation of multiple indexes. Lastly, CNN based anomaly detection identifies all the anomalies on multiple COVID-19 indexes with NLP-based explanations. A standalone decision support system was developed that implemented the presented method. This decision support system allows a strategic decision-maker to comprehend “when, where, and why there are fluctuations in COVID-19 related sentiments on a particular dimension”. Method was validated with Tweets from 15/072021 to 24/05/2022 resulting in automated generation of 5 COVID-19 indexes and 69 anomalies with explanations.

In summary, this method of anomaly detection on COVID-19 indexes presents:

• An explicit, transferable and reproducible procedure for detecting anomalies on multiple indexes of COVID-19 in multiple languages
• It helps a strategic decision maker to comprehend the root-causes of anomalies in COVID-19 related travel, vaccine, medical indexes
• The solution developed using the presented method allows evidence-based strategic decision-making COVID-19 crisis using AI, Deep Learning and NLP

## Graphical abstract

**Figure:**
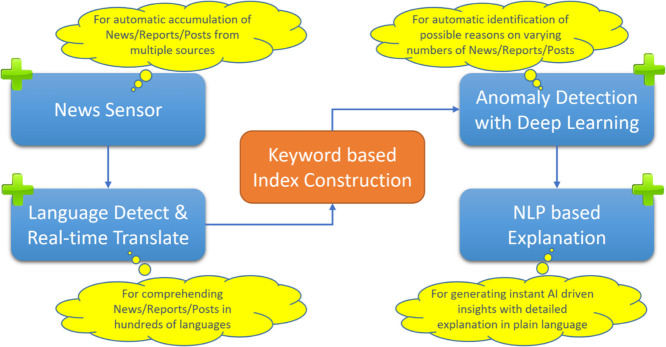

Specifications Table

**Table:** 

Subject Area	Computer Science
More specific subject area	Deep Learning
Method name	Anomaly detection on COVID-19 indexes
Name and reference of original method	Keyword based extraction of time-series COVID-19 indexes, • P. K. Narayan, B. N. Iyke and S. S. & Sharma, "New Measures of the COVID-19 Pandemic: A New Time-Series Dataset," Asian Economics Letters, vol. 2, no. 2, pp. 1–13, 2021 • F. K. Sufi and M. Alsulami, "AI-based automated extraction of location-oriented COVID-19 sentiments," Computers, Materials & Continua (CMC), vol. 72, no. 2, pp. 3631–3649, 2022. CNN based Anomaly Detection & Deep Learning • F. K. Sufi and M. Alsulami, "Automated Multidimensional Analysis of Global Events With Entity Detection, Sentiment Analysis and Anomaly Detection," IEEE Access, vol. 9, pp. 152449–152460, 2021. • F. Sufi and I. Khalil, "Automated Disaster Monitoring from Social Media Posts using AI based Location Intelligence and Sentiment Analysis," IEEE Transactions on Computational Social Systems, vol. Accepted (in Press), no. DOI: 10.1109/TCSS.2022.3157142, pp. 1–11, 2022. • F. K. Sufi and M. Alsulami, "Knowledge Discovery of Global Landslides Using Automated Machine Learning Algorithms," IEEE Access, vol. 9, 2021.
Resource availability	Input data (i.e., Effect.csv, medical.csv, travel.csv, uncertainity.csv, vaccine.csv), Microsoft Power BI Source File (i.e., COVID19_Anomaly.pbix), SQL Queries for Keyword based extraction (i.e., SQLQuery1.sql) and output COVID-19 Index file (i.e., COVID-19_Indexes.csv) are all publicly accessible from at https://github.com/DrSufi/COVID_Index_Anomaly

## Introduction

In Narayan and Iyke [1], researchers had proposed an innovative approach of using 327 keywords to extract multiple dimensions of COVID-19 like travel, vaccine, medical, uncertainty etc. These dimensions allowed the researchers to determine effects of specific COVID-19 related developments on financial and economic systems. By monitoring the frequencies of discussed topics from multiple news sources with the help of define keyword-sets (within each of the dimensions) as depicted in Narayan and Iyke [1], it is possible to detect fluctuations of sentiments among different dimensions of COVID-19. However, these fluctuations of sentiments would not automatically identify the underlying root-causes. For example, government decision on complete lockdown in Australia, might lower number of discussions and news related to travel which can easily be detected with travel index of Narayan and Iyke [1]. However, a strategic decision maker would not readily understand why there are lower number of travel related discussion in Australia. In another example, there might be in increase in COVID-19 vaccine related discussions in social media on a given day because of Anti-Vaccine related protests and negative sentiments as demonstrated in Sufi et al. [2]. Method demonstrated in Narayan and Iyke [1], would not flag the root-cause of these higher number of discussions because of Anti-Vax sentiments. Therefore, in this paper, we introduce application of Spectral Residual (SR) and Convolutional Neural Network (CNN) based deep learning to automatically detect and explain all the reasons for sentiment fluctuations on different dimensions COVID-19 from time-series data generated by solutions like [1]. Unlike the existing non-automated time-series analysis methods in Sharma [3], Devpura [4], Guru and Das [5], the proposed method automatically explains the root-causes of COVID-19 sentiment fluctuations in multiple dimensions harnessing the power of deep learning, NLP, and AI.

Using this updated method, a strategic decision maker can easily comprehend “when, where, and why there are fluctuations in COVID-19 related sentiment on a particular dimension”. Since the solution in Narayan and Iyke [1] produced time-series data on different dimensions, “when” questions could easily be answered. However, the “where” and “why” questions could not be answered by Narayan and Iyke [1]. Moreover, unlike previous studies, our novel approach works on all languages for all regions in the globe providing a compressive understanding on the effect COVID-19 globally.

Following are some the contributions of the presented method among many others:

• Understand which location (i.e., country, state, city etc.) is responsible for the fluctuations in COVID-19 related sentiments in multiple dimensions.
• Comprehend which languages (i.e., news in Hindi, Turkish, Chinese, Arabic etc.) are driving changes in the number of posts, reports, and news related COVID-19.
• Finding out the effect of COVID-19 related sentiments (i.e., Negative, Positive, Neutral) on the variations in COVID-19 associated news or posts.
• Instead of understanding reports, news, or social media posts in a single language (e.g., English as demonstrated in Narayan and Iyke [1]), the presented method understands in more than 110 different languages (recently demonstrated in Sufi et al. [2,[6], [7], [8], [9], [10], [11]]).
• Unlike obtaining news reports from only 45 sources, the presented method is capable of obtaining, aggregating, and analyzing reports from thousands of sources (e.g., 2397 different sources as demonstrated in our recent studies [[8], [9], [10]]).

## Method details

**Fig. 1:**
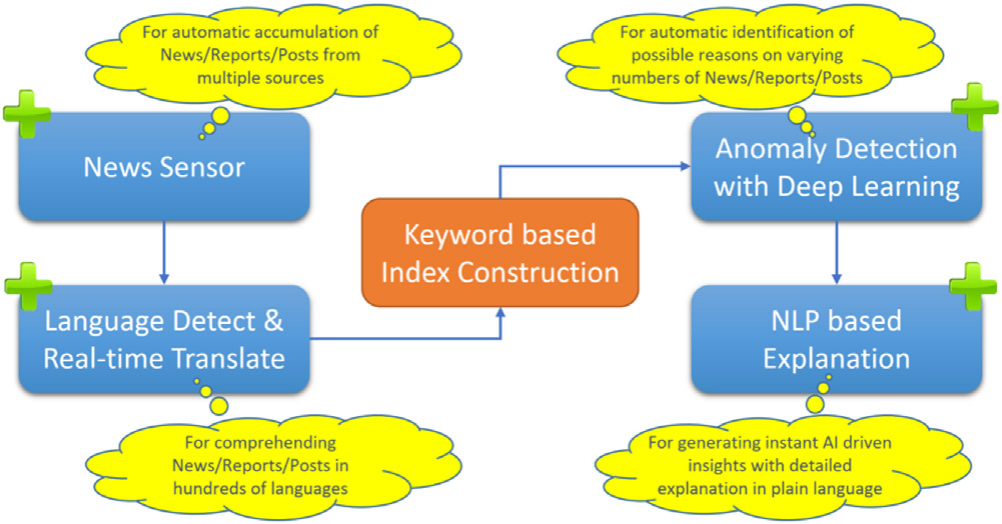
Conceptual Diagram of CNN & NLP on keyword-based extraction of COVID-19 indexes method.

This paper reports an update to the “Keyword based Index Construction” method described in Narayan and Iyke [1]. At first, we slightly modified the list of keywords presented for Medical, Travel, Vaccine, and Uncertainty indexes. Then, we added the following four modules, where each of these added modules introduces additional functionalities and improvements (as seen from Fig. 1):

(1) News Sensor.
(2) Language Detect & Real-time Translate.
(3) Anomaly Detection with Deep Learning (i.e., SR + CNN Algorithm).
(4) NLP based explanation.

(1) **News Sensor:**

News Sensor allowed Application Programming Interface (API) based connectivity to thousands of sources covering all major social media (e.g., Twitter, Facebook, Instagram, and YouTube), government websites (i.e., police websites, defense media websites, and foreign affairs websites), and the websites of national news agencies and of national and international TV/Radio channels, among others [[6], [7], [8], [9], [10], [11]]. In our most recent research work we had connected and automatically obtained news descriptions and posts from 2397 sources. Adding this, news sensor module with “keyword-based index construction” would allow us to accumulate COVID-19 related news, reports and posts from thousands of sources as opposed to only 45 sources depicted within [1].

(2) **Language Detect & Real-time Translate:**

Language, Detect & Translate Module allowed us to comprehend hundreds of languages from unrestricted global news sources, as opposed to only collecting news in English (as for the case in Narayan and Iyke [1]). Using Microsoft Cognitive Services’ Text Analytics API [12], the updated method can understand and analyze global COVID-19 related news and posts in hundreds of languages as demonstrated in our most recent research in [[6], [7], [8], [9], [10], [11]].

(3) **Deep Learning based Anomaly detection and NLP based explanation:**

Anomaly detection algorithms have recently been used for detecting and identifying anomalies on Time-Series data [[7], [8], [9], [10], [11], [12], [13], [14]]. Since, study in Narayan and Iyke [1] generated Time-Series data on a new set of COVID-19 index or dimension, adding Anomaly Detection algorithm would harness additional features like automatically detecting and highlighting sudden fluctuations in COVID-19 related news (in all the indexes reported within Narayan and Iyke [1]). Moreover, Anomaly detection can automatically find out the reason behind these fluctuations (i.e., answering questions like why, where and how). This in-depth analysis using deep learning techniques were not available within the methodology described in Narayan and Iyke [1].

The anomaly detector enhances line charts by automatically detecting anomalies within time-series data. It also provides explanations for the anomalies to facilitate root-cause analysis. In our most recent study, we have harnessed the anomaly detection algorithm to identify abnormal cases of landslides and obtain the root causes of these anomalies [13,14]. Moreover, we recognized abnormality and explained the abnormalities for disaster events using anomaly detection in social media [7,11]. Furthermore, we have classified anomalies on global events by monitoring 2397 global news sources and applying anomaly detection algorithms [8,9]. Before delving into the details of anomaly detection, we present the problem definition.

Problem 1: Given a sequence of real values, that is, $x=x_{1},\;x_{2},\;x_{3},\;\ldots ,\;x_{n},\;$the task of time-series anomaly detection is to produce an output sequence $y=y_{1},\;y_{2},\;y,\;\ldots ,\;y_{n},$ where $y_{i}\in \left\{0,1\right\}$ denotes whether $x_{i}$ is an anomaly point.

The implemented solution borrowed the SR from the visual saliency detection domain and then applied a CNN to the results produced by the SR model [15].

The SR algorithm consists of three major steps:

(1) Perform Fourier transform to obtain the log amplitude spectrum.
(2) Calculate the SR.
(3) Perform inverse Fourier transform, which transforms the sequence back to the spatial domain.
  (1) A(f)=Amplitude(f(x))
  (2) P(f)=Phrase(f(x))
  (3) L(f)=log(A(f))
  (4) $$AL\left(f\right)=h_{q}\left(f\right).L\left(f\right)$$
  (5) R(f)=L(f)−AL(f)
  (6) $$S\left(x\right)=\left|\left|f^{-1}\left(\exp\left(R\left(f\right)+iP\left(f\right)\right)\right)\right|\right|$$

where *f* and *f*^1^ denote the Fourier transform and inverse Fourier transform, respectively; *x* is the input sequence with shape *nX*1; *A(f)* is the amplitude spectrum of sequence ***x***; *P(f)* is the corresponding phase spectrum of sequence *x; L(f)* is the log representation of *A(f)*; and *AL(f)* is the average spectrum of *L(f)*, which can be approximated by convoluting the input sequence by *h_q_(f)*, where *h_q_(f)* is a *q × q* matrix defined as:

(7) $$h_{q}\left(f\right)=\frac{1}{q^{2}}\left[\begin{array}{c} \begin{array}{ccc} 1 & 1 & \ldots \\ 1 & 1 & \ldots \\ \cdots & \vdots & \ddots \end{array}\begin{array}{c} 1\\ 1\\ 1 \end{array}\\ \begin{array}{cc} 1 & 1 \end{array}\begin{array}{cc} \ldots & 1 \end{array} \end{array}\right]$$

*R(f)* is the SR, that is, the log spectrum *L(f)* minus the averaged log spectrum *AL(f)*. The SR serves as a compressed representation of the sequence, whereas the innovation part of the original sequence becomes more significant. Last, the sequence was transferred back to the spatial domain using an inverse Fourier transform. The resultant sequence *S(**x**)* is referred to as the saliency map [16]. The values of the anomaly points are calculated as follows:

(8) $$x=\left(\bar{x}+mean\right)\left(1+var\right).r+x$$

where $\bar{x}$ is the local average of the preceding points, mean and var are the mean and variance of all points within the current sliding window, and *r* ∼ *N* (0, 1) is randomly sampled. In this process, CNN is applied to the saliency map instead of to the raw input, thus increasing the efficiency of the overall process of anomaly detection [15,16].

The anomaly detection algorithm provides detailed explanations for all detected anomalies following the root-cause analysis performed by the AI services. In fact, we implement anomaly detection in three steps:

(1) Detect all the anomalies within the time series (i.e., any values that lie outside the threshold range).
(2) Identify the main drivers of these anomalies.
(3) Explain the results in a natural language (explanation of the root cause) using NLP [17].
(4) **Keyword based COVID-19 Index Construction:**

In our most recent research, we had automatically obtained thousands of COVID-19 related messages from social media using keywords like “COVID” or “CORONA” in up to 110 languages [6]. In another study, we have used multiple keywords (e.g., “Bushfire”, “Tornado”, “Flood”, “Hurricane”, “Landslide”, “Landfall”, “Earthquake” and others) to obtain real-time disaster related messages in multiple language from all parts of the globe (i.e., removing geographic barriers). In Narayan and Iyke [1], pre-defined group of keywords were used to target COVID-19 related news on different indexes like travel, medical, vaccine, and others. Using the list of keywords defined for multiple COVID-19 indexes as shown in Narayan and Iyke [1], we created our keyword-sets with minor modifications. As seen from Table 1, “Medical” dimension for this study uses 16 additional keywords compared to the solution demonstrated in Narayan and Iyke [1]. Similarly, for travel index, 6 more keywords were added on top the listed keywords in Narayan and Iyke [1]. 4 more keywords were added for “Vaccine” and another 4 more keywords were added for constructing “Uncertainty” dimension of COVID-19.

**Table 1:** Keywords used for extracting and generating different COVID-19 Indexes like Medical, Travel, Vaccine, Uncertainty, Effect etc.

Medical	Doctor, doctors, medicine, mask, medicines, nurse, nurses, health, care, recover, recovered, recoveries, recovering, recovers, recovery, symptom, symptoms, symptomatic, asymptomatic, infect, infected, infecting, infection, infectious, patient, patients, ambulance, ambulances, diagnose, diagnosed, diagnosis
Travel	Aircraft, aircrafts, airfare, airfares, airline, airplane, airplanes, airport, airports, attendant, attendants, pilot, pilots, plane, cabin, cabins, flight, flights, passengers, vacation, motel, motels, accommodation, holiday, holidaying, travel, travelling, travelled, travels
Vaccine	Vaccine, vaccines, pro-vaxxer, ani-vaxxer, vaxxer, vax
Uncertainty	Uncertain, uncertainty, risk, risked, risky, riskier, riskiest, risking, risks, riskiness, confusing, confuse, confused, confusion
COVID Effect	Virus, viruses, contagious, infect, infected, infections, infectious, infects, spread, spreads, outbreak, outbreaks, serious, patient, patients, emergencies, emergency, ambulance, asymptomatic, ambulances, symptom, symptoms, diagnose, diagnosed, symptomatic, isolate, isolated, isolating, isolation, quarantine, quarantined, quarantines, dead, deaths, death, die, died, shutdown, shutdowns, curfew

## Method Implementation

As mentioned before, the significant advantage introduced with our method of anomaly detections on COVID-19 indexes are following:

• Understanding News, Reports and Posts of more than 110 languages from multiple sources and multiple locations.
• User driven dynamic creation COVID-19 related indexes like effect, medical, travel, vaccine, uncertainty etc. with a click of a button.
• Fully automated detection of anomalies with COVID-19 indexes.
• Instant identifications of root-causes of these anomalies.
• Automated explanation generation of the root-causes of anomalies with NLP.

To realize the above benefits, we deployed a complete stand-alone solution with Microsoft Power BI, Microsoft Power Automate and associated technologies like Microsoft SQL Server, Microsoft Azure Cognitive Services etc. [12,18]. As seen from Table 2, capturing news and social media sources is performed with Microsoft Power Query, which is part of Microsoft Power BI [[6], [7], [8], [9], [10], [11]]. Moreover, Microsoft Power Automate is used for automatically capturing COVID-19 related news and posts as demonstrated in our recent study [6]. SQL Queries segregated COVID-19 index related posts in Microsoft SQL Server using the keywords shown in Table 1. The SQL script used for segregating these COVID-19 posts (for the individual indexes) are publicly available from my GitHub site [19] (i.e., the file with .sql extension). Moreover, the complete source code of the windows-based solution is located at [19] (i.e., the file with .pbix extension). Furthermore, the input files containing thousands of COVID-19 related Tweets (in .csv files) along with the output of COVID-19 indexes are all located in Sufi et al. [19]. A researcher can easily download the sources files and execute solution in desktop environment. The solution developed in this study can also be deployed in multiple platforms like iOS, Android etc. with the help of Microsoft Power BI services. Within our recent studies, we have successfully implemented Microsoft Power BI Services based Apps in multiple platforms like mobile, tablet and desktop [[6], [7], [8], [9], [10], [11], [12], [13], [14]]. These solutions allowed the strategic decision makers to perform evidence-based decisions while working from remote locations.

**Table 2:** Implementation of CNN & NLP on keyword-based extraction of COVID-19 indexes method.

**Features**	**Microsoft Power Query**	**Microsoft Power Automate**	**Microsoft SQL Server**	**Azure Cognitive Services**	**Microsoft Power BI Desktop**	**Microsoft Power BI Services**
Capturing News/ Social media posts	●	●				
Language Detect		●		●		
Translation		●		●		
Sentiment Analysis		●		●		
Entity Recognition		●		●		
COVID-19 Related Indexes			●		●	
Anomaly Detection (CNN)					●	
Root-Cause Explanation (NLP)					●	
Dashboard for Windows					●	●
Web based access						●
iOS App on Mobile/Tablet						●
Android App on Mobile/Tablet						●

The solution developed in this course of study, would allow a strategic decision maker to make evidence-based policy decisions on COVID-19 (in multiple dimensions like Travel, Vaccine, Medical etc.) using their own devices. Table 2 demonstrates technology components that supported different aspects of methods described within this paper.

## Method validation

At first, we systematically designed a method that applied CNN & NLP on keyword-based extraction of global COVID-19 indexes as seen from Fig. 1. With AI-based understanding of all languages, our innovative method could construct COVID-19 indexes in a much more comprehensive manner compared to previous methods in Narayan and Iyke [1]. Then, we implemented the designed method using various technological components (as demonstrated in Table 2). This allowed our solution to be deployed in multiple platforms like windows, iOS, and Android. Using the deployed solution [19], we tested and evaluated the presented method with Global Tweets data generated from 15 July 2021 to 24 May 2022. During these times Tweets in 62 different languages were captured (even though our system is capable of comprehending Tweets in well over 110 languages as demonstrated in [[6], [7], [8], [9], [10], [11]]). Moreover, there were more than 50K locations detected as the origins of these Tweets. Unlike [1], our method depicted in this study, retains location, language, along with other feature attributes, that could be used for detecting root-causes in anomalies of COVID-19 indexes.

(1) **Understanding of multiple languages & locations:**

**Fig. 2:**
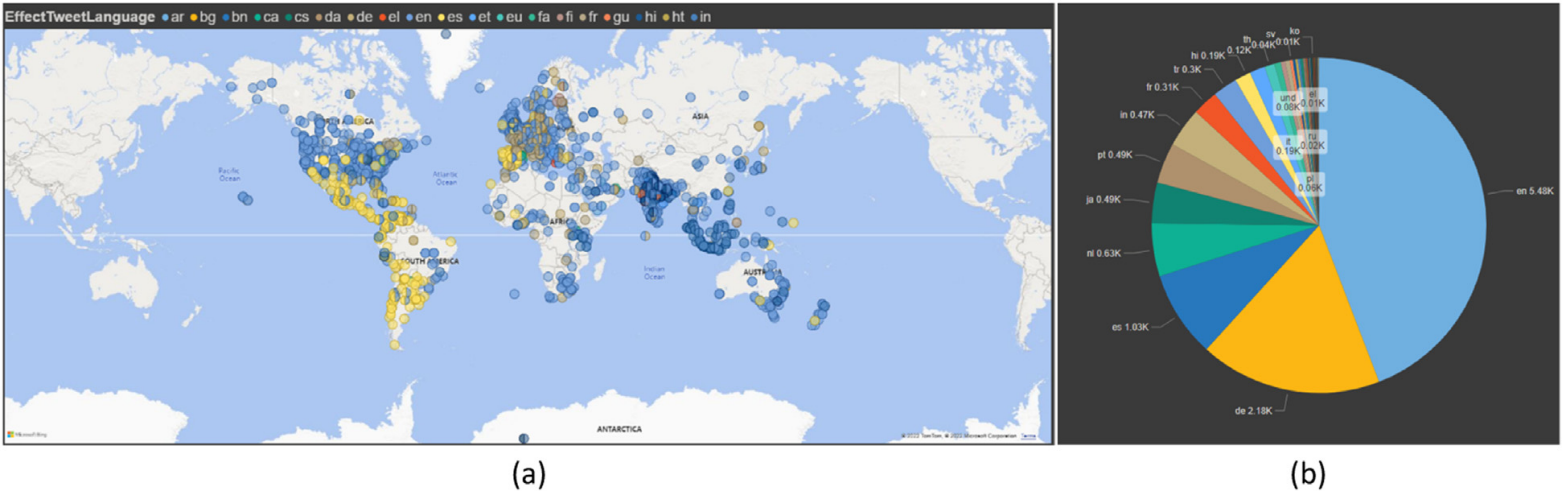
Tweet Locations and Languages for constructing Effect index. (a) Locations of COVID-19 effect related Tweets (b) Number of Effect related Tweets by different Languages.

**Fig. 3:**
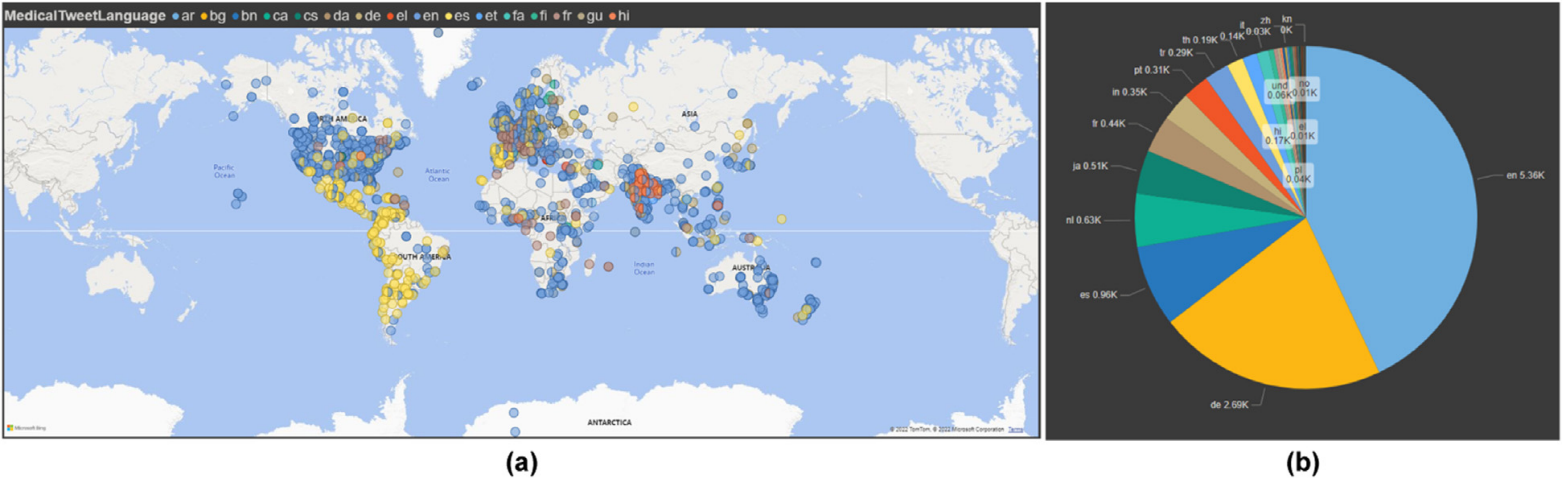
Tweet Locations and Languages for constructing Medical index. (a) Locations of Medical related Tweets (b) Number of Medical related Tweets by different Languages.

**Fig. 4:**
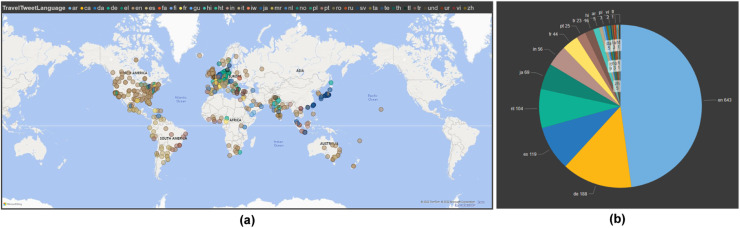
Tweet Locations and Languages for constructing Travel index. (a) Locations of Travel related Tweets (b) Number of Travel related Tweets by different Languages.

**Fig. 5:**
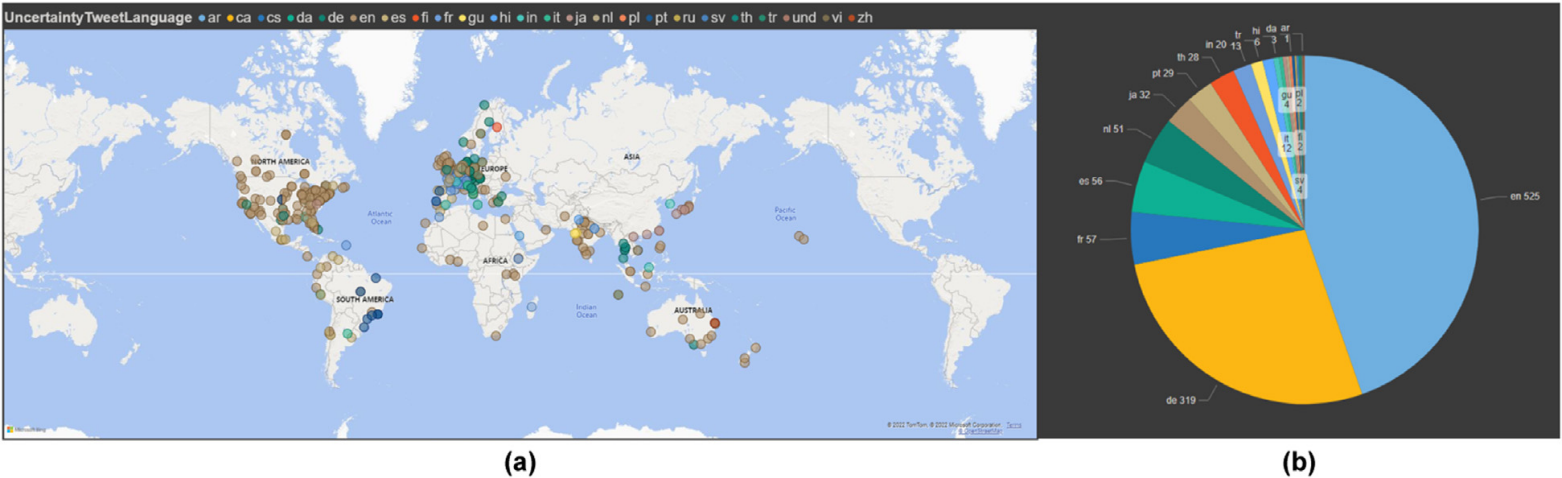
Tweet Locations and Languages for constructing Uncertainty index. (a) Locations of Uncertainty related Tweets (b) Number of Uncertainty related Tweets by different Languages.

**Fig. 6:**
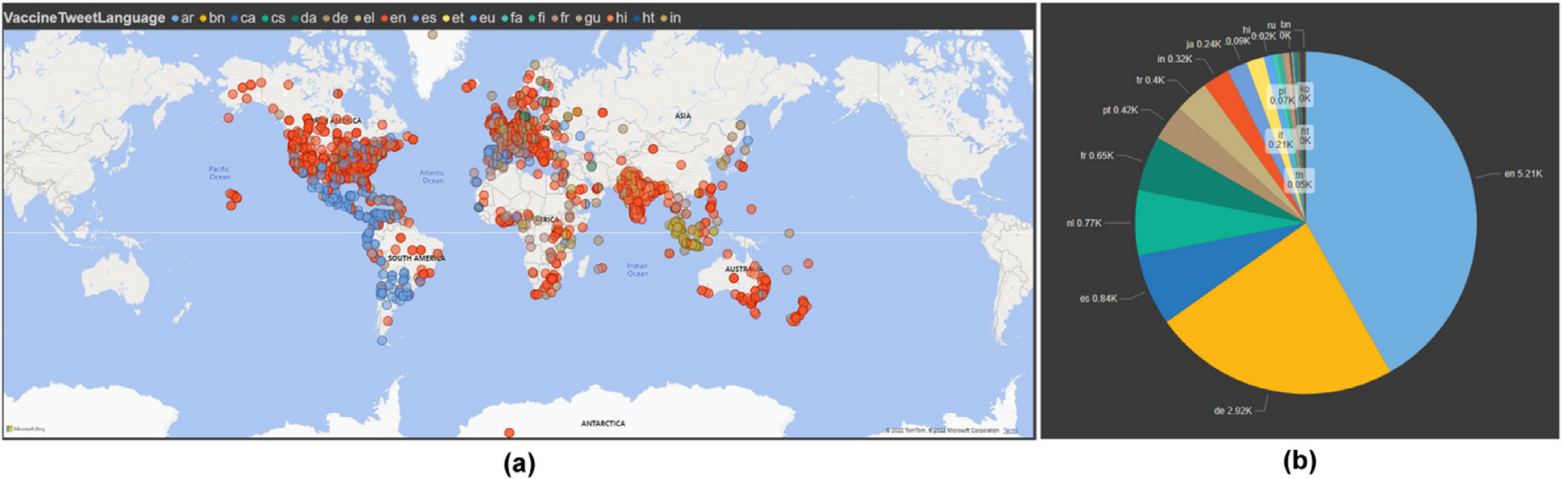
Tweet Locations and Languages for constructing Vaccine index. (a) Locations of Vaccine related Tweets (b) Number of Vaccine related Tweets by different Languages.

Figs. 2–6 shows the locations in Microsoft Bing Map along with number of Tweets per languages. As seen from these figures (i.e., Figs. 2–6), most of the Tweets were obtained in English (i.e., language code en), followed by German (i.e., language code de), Spanish (i.e., language code es), Dutch (i.e., language code nl), Japanese (i.e., language code ja), Portuguese (i.e., language code pt), and others. After obtaining the Tweets in different languages from different locations, they were translated and subsequently the keyword-based extraction process (demonstrated in Narayan and Iyke [1]) generated 5 COVID-19 related indexes (i.e., effect, medical, vaccine, travel, and uncertainty). Since the method depicted in this study is capable of comprehending News, posts, reports in multiple languages from multiple locations, the indexes generated in subsequent stages provide a more comprehensive and global representation of COVID-19 (i.e., as opposed to method in that only understood English posts from only 45 sources). It should be noted that even though our method evaluation section only uses posts from Twitter, “News Sensor” module (as shown in Fig. 1) can seamlessly retrieve News, reports, and posts from thousands of media sources (e.g., News websites, government sites, Facebook, Twitter, Instagram, Telegram etc.) in real-time as demonstrated in our recent studies [[8], [9], [10]].

(2) **COVID-19 Index Generation with Keyword extraction method:**

**Fig. 7:**
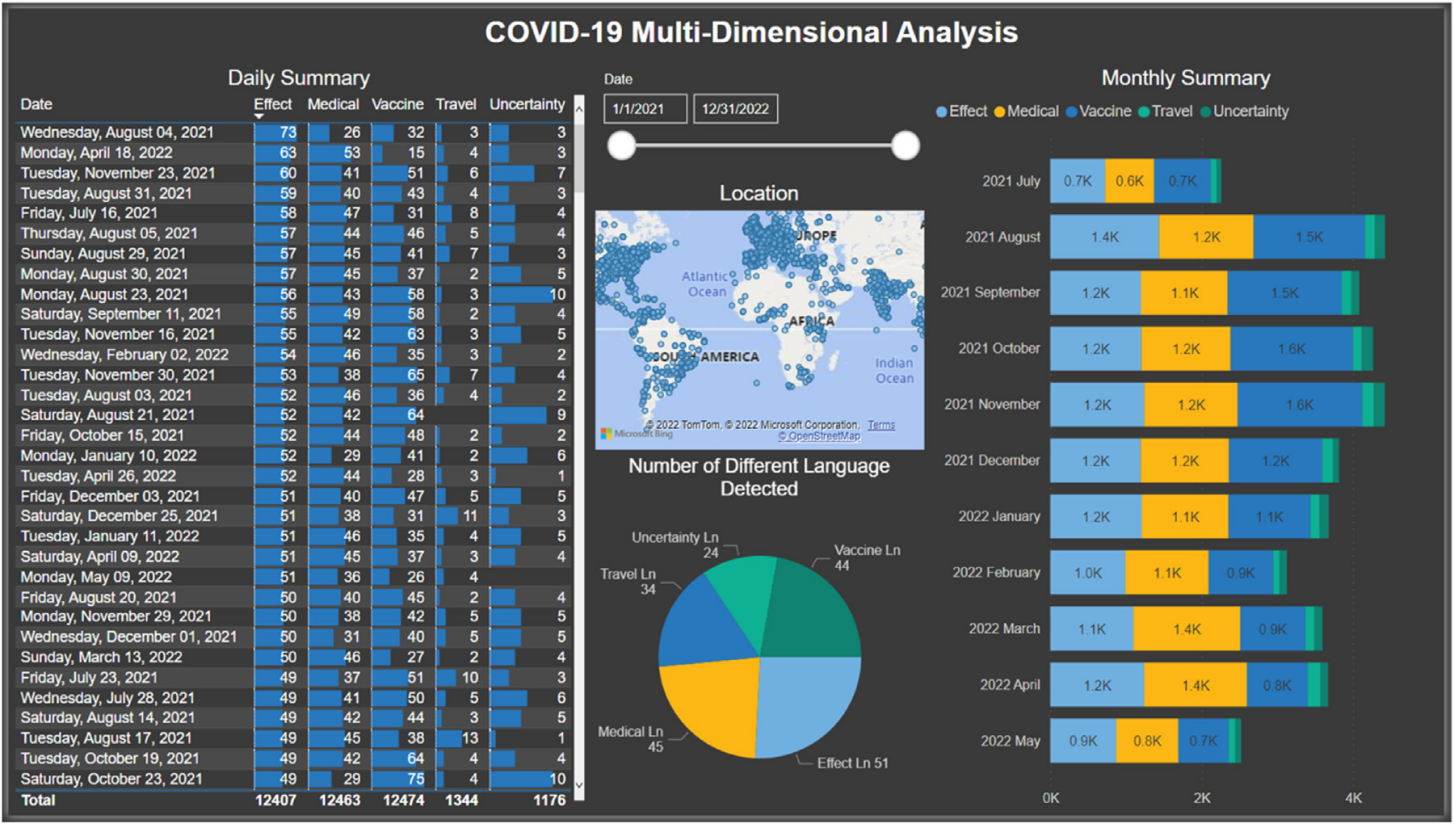
Multi-Dimensional Analysis of COVID-19 with our deployed windows solution [19].

Similar to the process described in Narayan and Iyke [1], time series data were generated by our method on 5 different COVID-19 related indexes as shown in Fig. 7 as well as within the table in Appendix). This extraction method used modified list of keywords as shown earlier in Table 1. Since the Tweets captured by “News Sensor” module (i.e., implemented in Microsoft Power Automate, and Microsoft Power Query) stored the Tweets in Microsoft SQL Server database, 5 different SQL queries were used to filter out only relevant COVID-19 related posts on effect, medical, travel, vaccine and uncertainty. These queries can be downloaded from Sufi et al. [19]. The deployed solution (i.e., [19]) can dynamically recreate COVID-19 related indexes like effect, medical, travel, vaccine, and uncertainty.

Fig. 7 shows the deployed Microsoft Power BI based solution [19]. Left side of Fig. 7 shows COVID-19 indexes (e.g., Medical, Vaccine, Travel, Uncertainty, Effect etc.) that were demonstrated in [1]. These indexes have been re-created with an updated set of keywords list (as seen in Table 1). The interactive interface shown in Fig. 7 allows a decision maker to select a date range and immediately index values are generated for that selected date range. Moreover, the summarized indexes could be seen by months (as it is shown in the right part of Fig. 7). The location (i.e., where the COVID-19 related posts were generated) could also be viewed in Map. Lastly, the pie chart shows that Effect index was calculated by comprehending 51 different languages, medical index was calculated from 45 different languages, Vaccine index was constructed based on understanding of 45 different languages. For construction of travel index Tweets of 34 different languages were used. Lastly, uncertainty related messages on COVID-19 were found in 24 different languages. It should be noted that with change of date range, all these values (i.e., of languages, locations, indexed) gets instantly recalculated and displayed in a highly interactive manner.

(3) **CNN based Anomaly detection and NLP based explanation:**

**Fig. 8:**
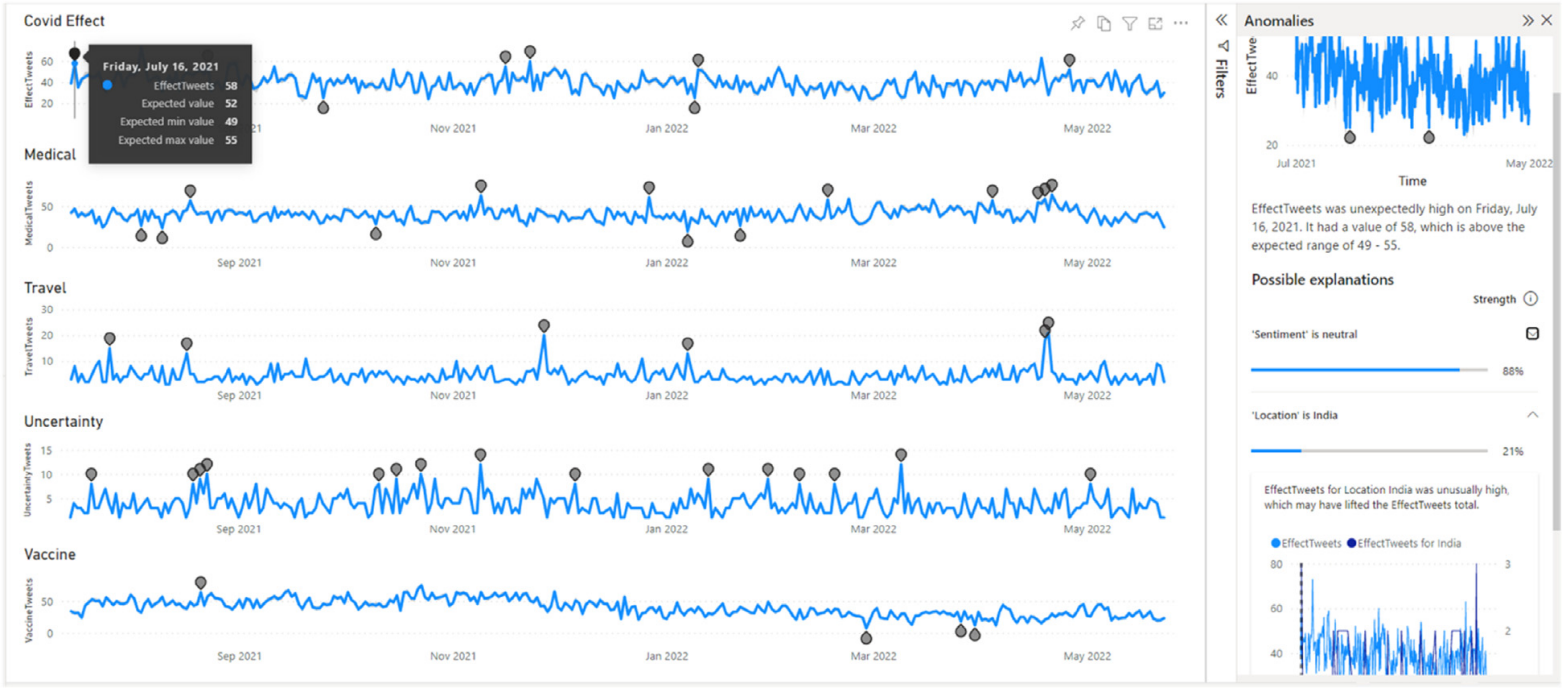
Anomaly detection algorithm tested on COVID Effect index of COVID-19 (75% Sensitivity).

The most interesting outcome of this study could be seen from Fig. 8, where all the anomalies for the calculated indexes (i.e., COVID-19 effect, medical, travel, vaccine, and uncertainty) are automatically detected by the newly introduced CNN based deep learning method. When a strategic user clicks any of these anomalies, the root-causes of these anomalies the instantly explained to the user in plain English language using NLP. As seen from Fig. 8, the user of the system clicked on an anomaly (i.e., Friday, July 16, 2021) on COVID Effect index. Immediately the possible explanations of that selected anomaly in displayed on the right side of the screen. As seen from Fig. 8, one of the reasons why that selected anomaly occurred is because there were higher numbers of COVID-19 related posts that originated from “India”. Since the presented system obtains social media posts from all over the world, in every possible language (i.e., supported by the social media platform), it can pinpoint which country, state, or city is responsible for the sudden outcry on COVID-19 situation. Without the application of deep learning methodology, manual identification the root causes from millions of messages, reports or news would be almost impossible.

**Fig. 9:**
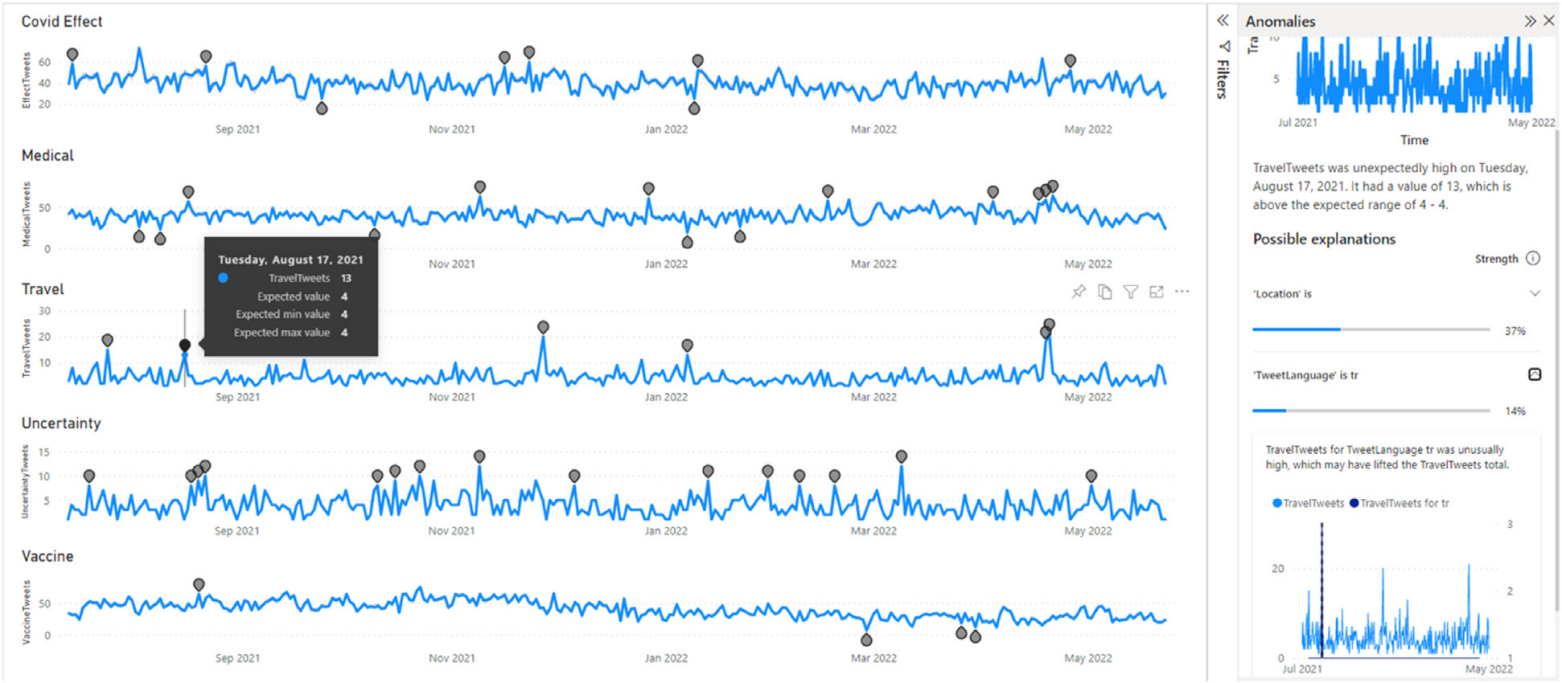
Anomaly detection algorithm tested on Travel index of COVID-19 (75% Sensitivity).

In another scenario, when the user clicked on the anomaly detected for Tuesday, August 17, 2021, root-cause analysis immediately performed CNN based deep learning (as seen from Fig. 9). As a result, the user is notified that on that day there were lots of Tweets generated by users whose location tags were disabled and on that day lots of COVID-19 related travel Tweets originated in “Turkish” language. This critical information provides an instant indication that on that particular day travel restriction and COVID-19 related outcry happened in Turkey, and as a result a noticeable surge was noticed on global COVID-19 Travel index.

**Fig. 10:**
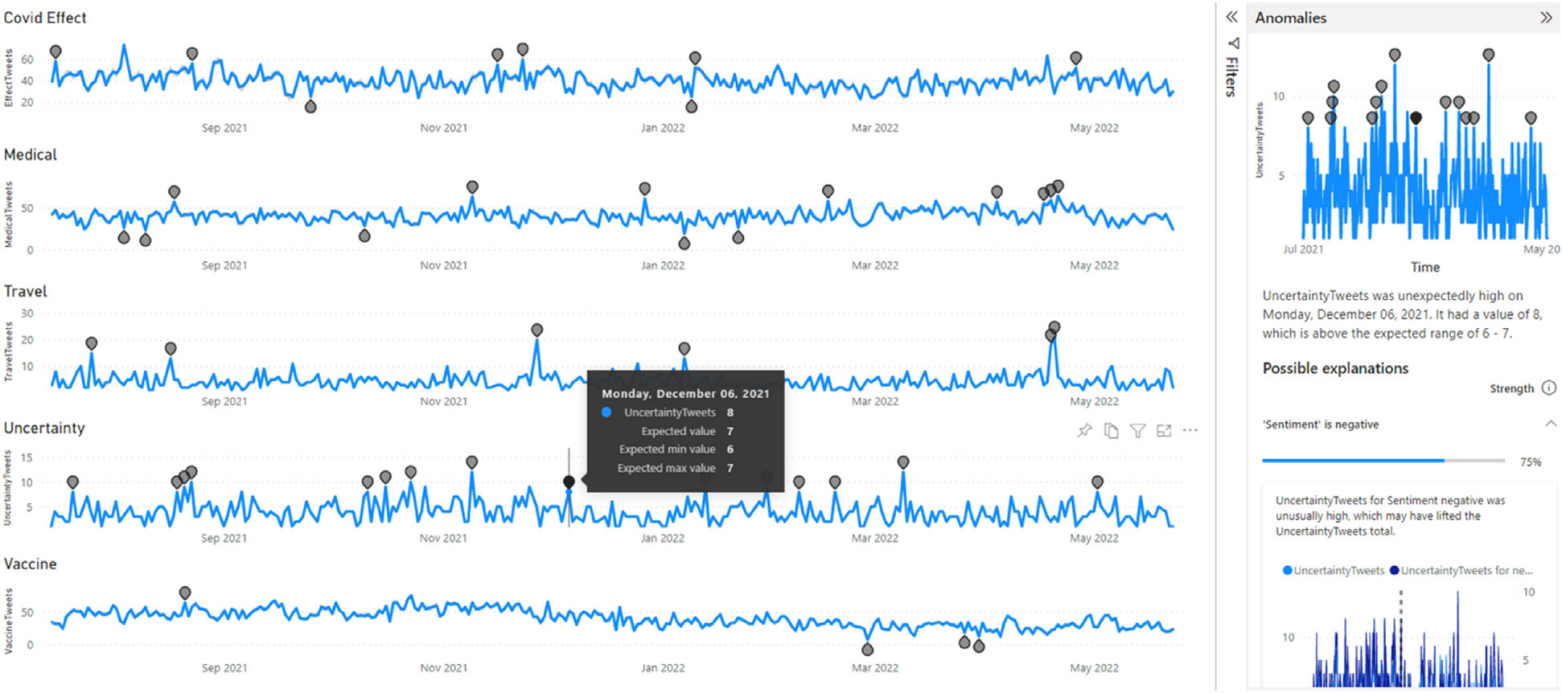
Anomaly detection algorithm tested on Uncertainty index of COVID-19 (75% Sensitivity).

In another scenario, the user of the system clicked on a particular anomaly on Monday, December 06, 2021 within Uncertainty index as seen from Fig. 10. Immediately, our innovative method found the possible explanation with corresponding strength (i.e., confidence of the explanation). For this instance, uncertainty level reached outside the tolerance level and the reason was on that particular day many uncertainties related posts had negative sentiment. This possible AI-driven explanation with 75% confidence informs the user that on that particular day high level of negativity was prevalent among the Tweet users (most likely because of global increase in COVID cases even after mass vaccination efforts by governments).

It should be highlighted that having AI-driven algorithms to instantly find out the root-causes (i.e., showing whether is it because of posts of a particular nation, particular language, particular sentiment, and all other variables) of anomalies present on COVID-19 indexes provide a significant improvement to the methods described in Narayan and Iyke [1].

In this research, we generated anomalies with varying anomaly sensitivities for COVID-19 effect, medical, travel, uncertainty, and vaccine indexes. With higher sensitivity percentage of the CNN-based anomaly detection algorithms, higher numbers of anomalies were identified. Figs. 8 to 10 reported anomalies with 75% sensitivity of implemented anomaly detection algorithm. Table 3 reports observations recorded from 75% to 100% sensitivities with an increment of 5% on all the five COVID-19 indexes. As seen from Table 3, Travel related anomalies were found to be more resistant to sensitivity changes as these anomalies lied the farthest from the corresponding travel index related tolerance values (i.e., expected minimum and expected maximum). As demonstrated in Table 3 with 100% sensitivity of the anomaly detection algorithms, in total 69 anomalies were recorded.

**Table 3:** Number of anomalies found for each of the COVID-19 Indexes with varying sensitivities of Anomaly detection algorithm.

Sensitivity	COVID Effect	Medical	Travel	Uncertainty	Vaccine
75%	1	1	6	10	0
80%	2	4	6	13	2
85%	6	5	6	13	2
90%	8	13	6	15	4
95%	14	18	6	16	10
100%	14	19	7	17	12

## Conclusion

In Narayan and Iyke [1], researchers demonstrated a method of generating new time-series indexes on COVID-19. These indexes allowed a new approach to analyze and measure the impacts of COVID-19 crisis on travel, medical, vaccination and many other domains. However, the method described in Narayan and Iyke [1], would require manual analysis to detect and explain anomalies. Moreover, time-series analysis showing the impact of COVID-19 on a range of factors like oil prices, currency exchange rates, stock markets shown in Shrama[3], Devpura [4], Guru and Das [5] are mostly non-automated.

**Fig. 11:**
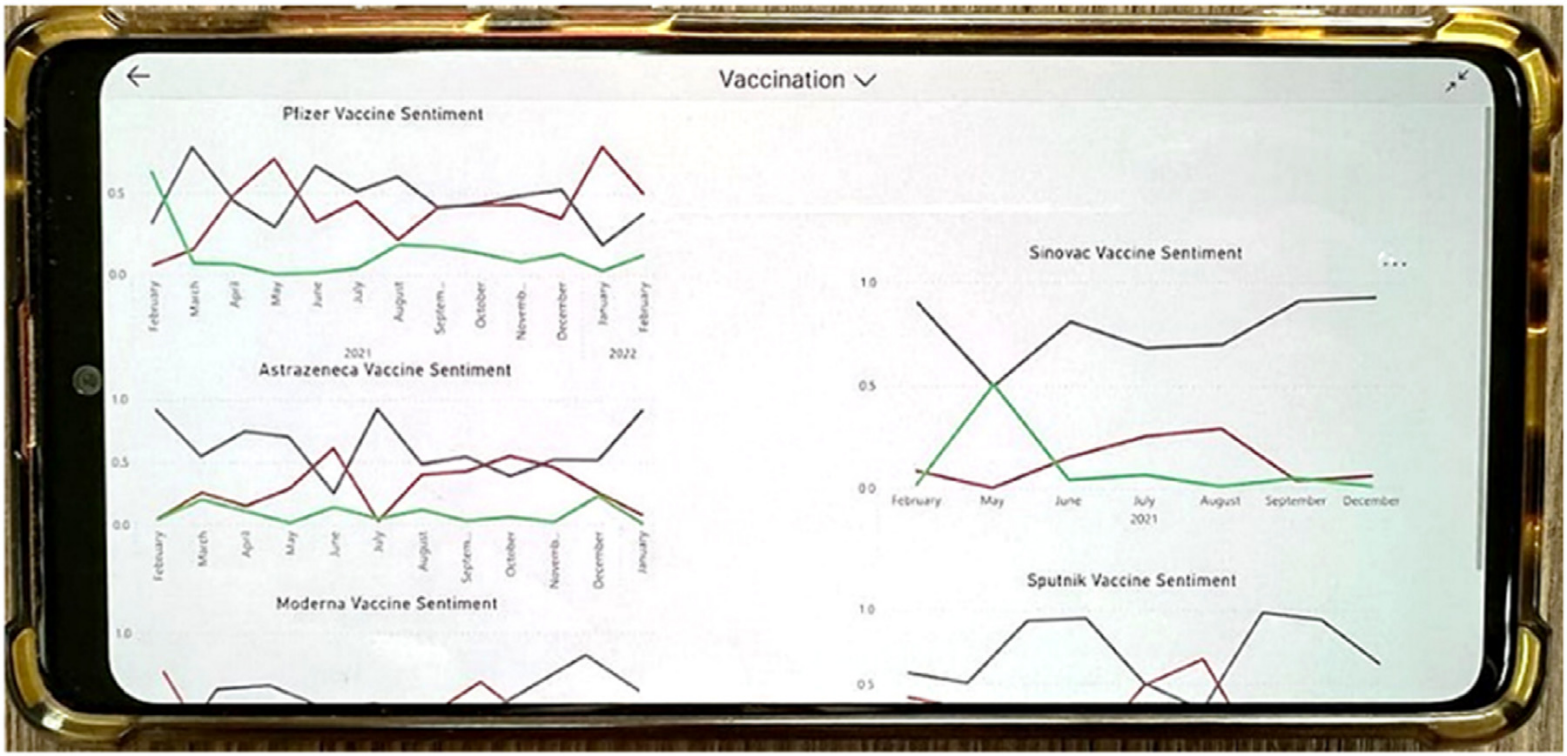
Pfizer, Astrazeneca, Moderna, Sinovac, and Sputnik vaccination index viewed in Samsung Galaxy Note 10 Lite Mobile (Deployed App running in Android Version 11).

This article demonstrated the detailed steps required to obtain AI-based root-causes on anomalies found in multiple COVID-19 indexes in complete automated manner. Moreover, the presented approach generates explanations of the root-causes in natural languages for the strategic decision-makers. Furthermore, as shown in Fig. 11, the innovative method was deployed in Mobile phones for strategic decision making. With methodological application of News sensor [[8], [9], [10]], language detection and translation [[6], [7], [8], [9], [10], [11]], keyword based extraction of COVID-19 indexes [[1], [2], [3], [4], [5], [6], [7]], CNN based Anomaly detection and explanation [[7], [8], [9],[11], [12], [13], [14]], this article demonstrated an automated multidimensional analytical capability of COVID-19 crisis.

One of the limitations of the presented method is that the creation of indexes is widely dependent the perspective of strategic analyst or decision maker. For example, a particular strategic analyst might be interested to on analyzing the vaccine index from a perspective of available mainstream COVID-19 vaccinations (e.g., US Pfizer, UK AstraZeneca, US Moderna, Chinese Sinovac, Russian Sputnik etc.). This implies updating the list of keywords for Vaccine index (as shown in Table 1) and adding keywords like Pfizer, AstraZeneca, Moderna, Sinovac, Sputnik etc. As seen from Fig. 11, adding these vaccine specific keywords would allow a strategic analyst to view and analyze individual vaccine specific indexes that might reveal geopolitical tensions arising from commercially available vaccines.

In another scenario, a strategic decision maker might want to critically analyze the vaccine index from a perspective of pro-vaccine and anti-vaccine sentiments. For these cases, keywords demonstrated in Table 1 for constructing vaccine index should be elaborated with “Ani-Vaxxer”, “Vaccine Hater”, “Vaccine Denier”, “COVID Scam” and others as demonstrated in Sufi et al. [2]. By maintaining a dynamic list of keywords (for constructing the indexes), the presented method can efficiently serve a wide range of strategic users for evidence-based decision-making.

**Supplementary material and/or Additional information:** All the source files (including the .pbix Microsoft Power BI solution, *.csv input Tweets for generating COVID-19 indexes, .csv output file on COVID-19 indexes) are located at https://github.com/DrSufi/COVID_Index_Anomaly). The method reported in this paper is an incremental development our previous research in social media analysis and news media analysis from 2397 sources. The source files pertaining to extracting COVID-19 and other natural disaster using keyword-based extraction techniques are located at IEEE's publicly accessible data repository [20,21]. The sources for files for automatic retrieval of News reports (i.e., from 2397 sources like BBC, CNN, New York times and others) and analyzing them with AI (as reported in [8], [9], [10]) is located at my GitHub site in Sufi [22].

## Declaration of Competing Interest

The author declares no known competing financial interests/ personal relationships that can influence this work.

## Data Availability

Data will be made available on request.

## Appendix Supplementary materials

Image, application 1
