# Supplementary material for: Automatic identification and explanation of root causes on COVID-19 index anomalies
Source: MethodsX. 2022 Dec 8;10:101960. doi: 10.1016/j.mex.2022.101960 (PMC9729591; doi:10.1016/j.mex.2022.101960)
Supplement: Supplementary file 1 [file mmc1.docx]

**Appendix:**

***Table.*** *COVID-19 Indexes for Effect, Medical, Vaccine, Travel, and Uncertainty from 15 July 2021 to 23 May 2022*

| Date | Effect | Medical | Vaccine | Travel | Uncertainty |
| --- | --- | --- | --- | --- | --- |
| 15/07/2021 | 39 | 42 | 34 | 3 | 1 |
| 16/07/2021 | 58 | 47 | 31 | 8 | 4 |
| 17/07/2021 | 35 | 37 | 32 | 2 | 3 |
| 18/07/2021 | 43 | 42 | 24 | 5 | 3 |
| 19/07/2021 | 45 | 38 | 43 | 2 | 2 |
| 20/07/2021 | 47 | 40 | 48 | 2 | 2 |
| 21/07/2021 | 45 | 45 | 53 | 5 | 8 |
| 22/07/2021 | 45 | 29 | 50 | 8 | 3 |
| 23/07/2021 | 49 | 37 | 51 | 10 | 3 |
| 24/07/2021 | 36 | 24 | 42 | 2 | 0 |
| 25/07/2021 | 31 | 29 | 56 | 2 | 7 |
| 26/07/2021 | 37 | 40 | 50 | 15 | 4 |
| 27/07/2021 | 39 | 48 | 44 | 3 | 2 |
| 28/07/2021 | 49 | 41 | 50 | 5 | 6 |
| 29/07/2021 | 48 | 41 | 46 | 2 | 1 |
| 30/07/2021 | 36 | 34 | 46 | 4 | 4 |
| 31/07/2021 | 44 | 32 | 48 | 4 | 3 |
| 01/08/2021 | 48 | 39 | 60 | 8 | 3 |
| 02/08/2021 | 47 | 39 | 54 | 6 | 6 |
| 03/08/2021 | 52 | 46 | 36 | 4 | 2 |
| 04/08/2021 | 73 | 26 | 32 | 3 | 3 |
| 05/08/2021 | 57 | 44 | 46 | 5 | 4 |
| 06/08/2021 | 41 | 32 | 53 | 5 | 5 |
| 07/08/2021 | 43 | 45 | 41 | 8 | 3 |
| 08/08/2021 | 45 | 36 | 46 | 3 | 3 |
| 09/08/2021 | 45 | 37 | 49 | 2 | 3 |
| 10/08/2021 | 31 | 23 | 57 | 8 | 1 |
| 11/08/2021 | 39 | 40 | 43 | 1 | 4 |
| 12/08/2021 | 46 | 41 | 46 | 1 | 2 |
| 13/08/2021 | 39 | 45 | 51 | 7 | 4 |
| 14/08/2021 | 49 | 42 | 44 | 3 | 5 |
| 15/08/2021 | 32 | 32 | 46 | 3 | 5 |
| 16/08/2021 | 46 | 44 | 40 | 8 | 4 |
| 17/08/2021 | 49 | 45 | 38 | 13 | 1 |
| 18/08/2021 | 48 | 57 | 49 | 5 | 3 |
| 19/08/2021 | 47 | 46 | 43 | 5 | 8 |
| 20/08/2021 | 50 | 40 | 45 | 2 | 4 |
| 21/08/2021 | 52 | 42 | 64 | 0 | 9 |
| 22/08/2021 | 47 | 41 | 43 | 2 | 6 |
| 23/08/2021 | 56 | 43 | 58 | 3 | 10 |
| 24/08/2021 | 32 | 30 | 62 | 3 | 3 |
| 25/08/2021 | 39 | 42 | 55 | 4 | 3 |
| 26/08/2021 | 40 | 45 | 54 | 4 | 5 |
| 27/08/2021 | 33 | 33 | 52 | 2 | 5 |
| 28/08/2021 | 39 | 44 | 42 | 4 | 6 |
| 29/08/2021 | 57 | 45 | 41 | 7 | 3 |
| 30/08/2021 | 57 | 45 | 37 | 2 | 5 |
| 31/08/2021 | 59 | 40 | 43 | 4 | 3 |
| 01/09/2021 | 41 | 42 | 52 | 3 | 1 |
| 02/09/2021 | 37 | 42 | 39 | 5 | 3 |
| 03/09/2021 | 47 | 39 | 54 | 1 | 6 |
| 04/09/2021 | 44 | 44 | 52 | 4 | 3 |
| 05/09/2021 | 34 | 31 | 39 | 3 | 5 |
| 06/09/2021 | 35 | 32 | 52 | 1 | 8 |
| 07/09/2021 | 38 | 38 | 40 | 2 | 2 |
| 08/09/2021 | 45 | 40 | 46 | 4 | 3 |
| 09/09/2021 | 40 | 42 | 51 | 4 | 7 |
| 10/09/2021 | 44 | 32 | 54 | 9 | 0 |
| 11/09/2021 | 55 | 49 | 58 | 2 | 4 |
| 12/09/2021 | 40 | 36 | 53 | 9 | 4 |
| 13/09/2021 | 43 | 43 | 56 | 3 | 3 |
| 14/09/2021 | 42 | 40 | 63 | 5 | 4 |
| 15/09/2021 | 47 | 45 | 67 | 5 | 0 |
| 16/09/2021 | 47 | 37 | 57 | 6 | 3 |
| 17/09/2021 | 44 | 42 | 62 | 4 | 1 |
| 18/09/2021 | 27 | 39 | 45 | 4 | 4 |
| 19/09/2021 | 27 | 43 | 38 | 4 | 5 |
| 20/09/2021 | 25 | 39 | 48 | 11 | 1 |
| 21/09/2021 | 36 | 34 | 55 | 5 | 2 |
| 22/09/2021 | 48 | 44 | 40 | 3 | 2 |
| 23/09/2021 | 40 | 38 | 39 | 3 | 3 |
| 24/09/2021 | 44 | 29 | 39 | 4 | 6 |
| 25/09/2021 | 25 | 30 | 46 | 4 | 4 |
| 26/09/2021 | 40 | 39 | 44 | 5 | 3 |
| 27/09/2021 | 43 | 34 | 45 | 7 | 2 |
| 28/09/2021 | 37 | 38 | 58 | 2 | 5 |
| 29/09/2021 | 43 | 36 | 54 | 3 | 5 |
| 30/09/2021 | 39 | 32 | 57 | 5 | 6 |
| 01/10/2021 | 40 | 46 | 47 | 2 | 5 |
| 02/10/2021 | 47 | 45 | 64 | 1 | 7 |
| 03/10/2021 | 39 | 44 | 52 | 5 | 4 |
| 04/10/2021 | 44 | 38 | 48 | 5 | 3 |
| 05/10/2021 | 30 | 36 | 37 | 4 | 4 |
| 06/10/2021 | 41 | 40 | 42 | 7 | 4 |
| 07/10/2021 | 35 | 44 | 49 | 2 | 4 |
| 08/10/2021 | 38 | 37 | 43 | 5 | 1 |
| 09/10/2021 | 38 | 38 | 45 | 6 | 2 |
| 10/10/2021 | 35 | 28 | 43 | 4 | 7 |
| 11/10/2021 | 35 | 44 | 46 | 6 | 8 |
| 12/10/2021 | 43 | 38 | 41 | 5 | 2 |
| 13/10/2021 | 34 | 37 | 42 | 6 | 7 |
| 14/10/2021 | 33 | 38 | 52 | 4 | 5 |
| 15/10/2021 | 52 | 44 | 48 | 2 | 2 |
| 16/10/2021 | 38 | 32 | 59 | 4 | 9 |
| 17/10/2021 | 36 | 34 | 35 | 3 | 2 |
| 18/10/2021 | 40 | 27 | 64 | 1 | 5 |
| 19/10/2021 | 49 | 42 | 64 | 4 | 4 |
| 20/10/2021 | 47 | 50 | 55 | 3 | 6 |
| 21/10/2021 | 30 | 33 | 54 | 3 | 8 |
| 22/10/2021 | 31 | 34 | 69 | 2 | 5 |
| 23/10/2021 | 49 | 29 | 75 | 4 | 10 |
| 24/10/2021 | 40 | 31 | 56 | 2 | 7 |
| 25/10/2021 | 24 | 30 | 52 | 5 | 2 |
| 26/10/2021 | 36 | 44 | 63 | 4 | 4 |
| 27/10/2021 | 37 | 43 | 54 | 2 | 9 |
| 28/10/2021 | 45 | 43 | 56 | 2 | 4 |
| 29/10/2021 | 34 | 39 | 57 | 7 | 3 |
| 30/10/2021 | 33 | 33 | 60 | 3 | 5 |
| 31/10/2021 | 49 | 39 | 43 | 4 | 5 |
| 01/11/2021 | 34 | 42 | 47 | 4 | 4 |
| 02/11/2021 | 36 | 44 | 64 | 10 | 4 |
| 03/11/2021 | 39 | 35 | 61 | 2 | 4 |
| 04/11/2021 | 37 | 43 | 63 | 7 | 7 |
| 05/11/2021 | 29 | 33 | 61 | 5 | 7 |
| 06/11/2021 | 39 | 51 | 51 | 3 | 6 |
| 07/11/2021 | 28 | 37 | 57 | 5 | 2 |
| 08/11/2021 | 36 | 46 | 46 | 8 | 2 |
| 09/11/2021 | 43 | 63 | 64 | 4 | 12 |
| 10/11/2021 | 44 | 40 | 54 | 3 | 5 |
| 11/11/2021 | 47 | 48 | 55 | 7 | 7 |
| 12/11/2021 | 47 | 46 | 57 | 8 | 6 |
| 13/11/2021 | 29 | 35 | 63 | 2 | 2 |
| 14/11/2021 | 40 | 35 | 57 | 2 | 4 |
| 15/11/2021 | 42 | 46 | 59 | 0 | 2 |
| 16/11/2021 | 55 | 42 | 63 | 3 | 5 |
| 17/11/2021 | 30 | 47 | 50 | 3 | 5 |
| 18/11/2021 | 49 | 38 | 55 | 2 | 2 |
| 19/11/2021 | 42 | 51 | 63 | 1 | 3 |
| 20/11/2021 | 42 | 32 | 52 | 3 | 2 |
| 21/11/2021 | 45 | 32 | 61 | 2 | 7 |
| 22/11/2021 | 40 | 26 | 62 | 6 | 5 |
| 23/11/2021 | 60 | 41 | 51 | 6 | 7 |
| 24/11/2021 | 32 | 32 | 48 | 7 | 5 |
| 25/11/2021 | 47 | 47 | 56 | 3 | 9 |
| 26/11/2021 | 33 | 44 | 42 | 11 | 7 |
| 27/11/2021 | 49 | 42 | 43 | 20 | 5 |
| 28/11/2021 | 46 | 38 | 33 | 6 | 1 |
| 29/11/2021 | 50 | 38 | 42 | 5 | 5 |
| 30/11/2021 | 53 | 38 | 65 | 7 | 4 |
| 01/12/2021 | 50 | 31 | 40 | 5 | 5 |
| 02/12/2021 | 44 | 44 | 36 | 8 | 0 |
| 03/12/2021 | 51 | 40 | 47 | 5 | 5 |
| 04/12/2021 | 45 | 32 | 43 | 1 | 2 |
| 05/12/2021 | 29 | 36 | 37 | 3 | 5 |
| 06/12/2021 | 33 | 35 | 51 | 2 | 8 |
| 07/12/2021 | 28 | 36 | 47 | 4 | 2 |
| 08/12/2021 | 47 | 35 | 30 | 5 | 3 |
| 09/12/2021 | 48 | 43 | 45 | 6 | 0 |
| 10/12/2021 | 44 | 46 | 50 | 4 | 2 |
| 11/12/2021 | 37 | 37 | 47 | 4 | 5 |
| 12/12/2021 | 36 | 31 | 41 | 3 | 5 |
| 13/12/2021 | 34 | 31 | 52 | 4 | 2 |
| 14/12/2021 | 28 | 27 | 46 | 1 | 2 |
| 15/12/2021 | 37 | 48 | 46 | 5 | 0 |
| 16/12/2021 | 36 | 44 | 36 | 5 | 3 |
| 17/12/2021 | 25 | 34 | 37 | 4 | 1 |
| 18/12/2021 | 41 | 43 | 49 | 9 | 6 |
| 19/12/2021 | 36 | 38 | 22 | 4 | 1 |
| 20/12/2021 | 33 | 42 | 57 | 6 | 1 |
| 21/12/2021 | 31 | 31 | 29 | 3 | 4 |
| 22/12/2021 | 38 | 28 | 41 | 2 | 3 |
| 23/12/2021 | 34 | 43 | 42 | 3 | 3 |
| 24/12/2021 | 35 | 36 | 21 | 6 | 0 |
| 25/12/2021 | 51 | 38 | 31 | 11 | 3 |
| 26/12/2021 | 42 | 30 | 32 | 3 | 1 |
| 27/12/2021 | 40 | 61 | 39 | 2 | 1 |
| 28/12/2021 | 42 | 39 | 40 | 5 | 4 |
| 29/12/2021 | 43 | 37 | 32 | 6 | 2 |
| 30/12/2021 | 39 | 38 | 41 | 5 | 2 |
| 31/12/2021 | 36 | 34 | 24 | 7 | 2 |
| 01/01/2022 | 39 | 40 | 22 | 3 | 1 |
| 02/01/2022 | 44 | 35 | 32 | 3 | 1 |
| 03/01/2022 | 45 | 42 | 31 | 6 | 4 |
| 04/01/2022 | 38 | 35 | 48 | 9 | 2 |
| 05/01/2022 | 41 | 32 | 34 | 5 | 5 |
| 06/01/2022 | 44 | 44 | 28 | 3 | 3 |
| 07/01/2022 | 29 | 19 | 35 | 13 | 3 |
| 08/01/2022 | 38 | 36 | 38 | 7 | 7 |
| 09/01/2022 | 25 | 32 | 32 | 2 | 4 |
| 10/01/2022 | 52 | 29 | 41 | 2 | 6 |
| 11/01/2022 | 51 | 46 | 35 | 4 | 5 |
| 12/01/2022 | 48 | 35 | 25 | 4 | 3 |
| 13/01/2022 | 43 | 38 | 31 | 6 | 9 |
| 14/01/2022 | 40 | 30 | 29 | 3 | 1 |
| 15/01/2022 | 35 | 26 | 33 | 3 | 3 |
| 16/01/2022 | 46 | 38 | 40 | 3 | 0 |
| 17/01/2022 | 36 | 43 | 46 | 7 | 1 |
| 18/01/2022 | 41 | 31 | 30 | 4 | 3 |
| 19/01/2022 | 35 | 43 | 27 | 3 | 3 |
| 20/01/2022 | 34 | 40 | 33 | 3 | 5 |
| 21/01/2022 | 46 | 46 | 33 | 2 | 5 |
| 22/01/2022 | 31 | 26 | 41 | 0 | 5 |
| 23/01/2022 | 41 | 47 | 38 | 4 | 6 |
| 24/01/2022 | 36 | 32 | 35 | 3 | 4 |
| 25/01/2022 | 40 | 49 | 32 | 4 | 3 |
| 26/01/2022 | 31 | 38 | 31 | 1 | 4 |
| 27/01/2022 | 29 | 39 | 44 | 2 | 5 |
| 28/01/2022 | 37 | 46 | 37 | 3 | 6 |
| 29/01/2022 | 33 | 36 | 32 | 1 | 5 |
| 30/01/2022 | 44 | 44 | 49 | 3 | 9 |
| 31/01/2022 | 36 | 31 | 38 | 3 | 3 |
| 01/02/2022 | 47 | 36 | 33 | 4 | 4 |
| 02/02/2022 | 54 | 46 | 35 | 3 | 2 |
| 03/02/2022 | 47 | 39 | 43 | 2 | 3 |
| 04/02/2022 | 36 | 31 | 43 | 5 | 2 |
| 05/02/2022 | 38 | 33 | 36 | 5 | 0 |
| 06/02/2022 | 28 | 32 | 27 | 2 | 4 |
| 07/02/2022 | 46 | 44 | 21 | 4 | 3 |
| 08/02/2022 | 37 | 52 | 37 | 2 | 8 |
| 09/02/2022 | 34 | 41 | 22 | 2 | 4 |
| 10/02/2022 | 28 | 33 | 26 | 4 | 2 |
| 11/02/2022 | 36 | 47 | 39 | 6 | 6 |
| 12/02/2022 | 34 | 39 | 38 | 1 | 3 |
| 13/02/2022 | 34 | 36 | 29 | 4 | 2 |
| 14/02/2022 | 39 | 34 | 31 | 7 | 4 |
| 15/02/2022 | 25 | 37 | 32 | 1 | 2 |
| 16/02/2022 | 33 | 58 | 37 | 6 | 2 |
| 17/02/2022 | 40 | 35 | 31 | 1 | 2 |
| 18/02/2022 | 28 | 39 | 38 | 1 | 8 |
| 19/02/2022 | 40 | 48 | 37 | 2 | 5 |
| 20/02/2022 | 39 | 42 | 25 | 2 | 2 |
| 21/02/2022 | 34 | 45 | 27 | 1 | 3 |
| 22/02/2022 | 33 | 59 | 33 | 1 | 4 |
| 23/02/2022 | 36 | 38 | 29 | 6 | 4 |
| 24/02/2022 | 30 | 32 | 27 | 3 | 6 |
| 25/02/2022 | 23 | 28 | 26 | 1 | 1 |
| 26/02/2022 | 36 | 29 | 24 | 3 | 1 |
| 27/02/2022 | 33 | 31 | 7 | 5 | 3 |
| 28/02/2022 | 25 | 35 | 20 | 4 | 5 |
| 01/03/2022 | 24 | 46 | 33 | 5 | 5 |
| 02/03/2022 | 27 | 54 | 39 | 6 | 7 |
| 03/03/2022 | 28 | 55 | 24 | 3 | 4 |
| 04/03/2022 | 36 | 44 | 23 | 0 | 2 |
| 05/03/2022 | 42 | 41 | 22 | 3 | 1 |
| 06/03/2022 | 38 | 35 | 35 | 6 | 2 |
| 07/03/2022 | 43 | 46 | 27 | 1 | 5 |
| 08/03/2022 | 30 | 37 | 22 | 5 | 4 |
| 09/03/2022 | 26 | 50 | 30 | 4 | 12 |
| 10/03/2022 | 26 | 47 | 30 | 8 | 2 |
| 11/03/2022 | 42 | 44 | 19 | 3 | 6 |
| 12/03/2022 | 43 | 46 | 13 | 4 | 2 |
| 13/03/2022 | 50 | 46 | 27 | 2 | 4 |
| 14/03/2022 | 30 | 52 | 27 | 5 | 5 |
| 15/03/2022 | 40 | 46 | 31 | 8 | 2 |
| 16/03/2022 | 35 | 49 | 33 | 3 | 1 |
| 17/03/2022 | 27 | 43 | 31 | 4 | 4 |
| 18/03/2022 | 46 | 58 | 33 | 5 | 2 |
| 19/03/2022 | 36 | 50 | 34 | 0 | 4 |
| 20/03/2022 | 37 | 37 | 30 | 1 | 4 |
| 21/03/2022 | 26 | 41 | 34 | 2 | 2 |
| 22/03/2022 | 39 | 44 | 30 | 1 | 3 |
| 23/03/2022 | 39 | 50 | 30 | 7 | 3 |
| 24/03/2022 | 37 | 45 | 26 | 2 | 3 |
| 25/03/2022 | 42 | 50 | 33 | 3 | 1 |
| 26/03/2022 | 28 | 47 | 18 | 2 | 2 |
| 27/03/2022 | 41 | 40 | 34 | 8 | 6 |
| 28/03/2022 | 39 | 43 | 22 | 4 | 3 |
| 29/03/2022 | 37 | 43 | 27 | 2 | 1 |
| 30/03/2022 | 27 | 30 | 12 | 3 | 3 |
| 31/03/2022 | 36 | 54 | 33 | 4 | 5 |
| 01/04/2022 | 41 | 52 | 20 | 4 | 3 |
| 02/04/2022 | 38 | 46 | 22 | 7 | 3 |
| 03/04/2022 | 41 | 42 | 19 | 4 | 2 |
| 04/04/2022 | 33 | 57 | 23 | 7 | 5 |
| 05/04/2022 | 42 | 41 | 12 | 4 | 1 |
| 06/04/2022 | 40 | 43 | 33 | 9 | 2 |
| 07/04/2022 | 40 | 37 | 44 | 3 | 1 |
| 08/04/2022 | 32 | 45 | 39 | 3 | 7 |
| 09/04/2022 | 51 | 45 | 37 | 3 | 4 |
| 10/04/2022 | 41 | 30 | 23 | 8 | 4 |
| 11/04/2022 | 42 | 36 | 16 | 2 | 1 |
| 12/04/2022 | 49 | 32 | 25 | 6 | 5 |
| 13/04/2022 | 36 | 38 | 25 | 7 | 3 |
| 14/04/2022 | 42 | 44 | 16 | 6 | 3 |
| 15/04/2022 | 43 | 41 | 26 | 8 | 2 |
| 16/04/2022 | 39 | 31 | 24 | 9 | 7 |
| 17/04/2022 | 42 | 55 | 20 | 3 | 0 |
| 18/04/2022 | 63 | 53 | 15 | 4 | 3 |
| 19/04/2022 | 44 | 59 | 21 | 18 | 2 |
| 20/04/2022 | 28 | 45 | 23 | 21 | 3 |
| 21/04/2022 | 39 | 64 | 30 | 6 | 2 |
| 22/04/2022 | 44 | 55 | 25 | 5 | 6 |
| 23/04/2022 | 41 | 48 | 30 | 2 | 3 |
| 24/04/2022 | 47 | 45 | 29 | 5 | 6 |
| 25/04/2022 | 45 | 55 | 33 | 3 | 7 |
| 26/04/2022 | 52 | 44 | 28 | 3 | 1 |
| 27/04/2022 | 32 | 46 | 25 | 3 | 2 |
| 28/04/2022 | 41 | 52 | 32 | 1 | 3 |
| 29/04/2022 | 42 | 40 | 42 | 5 | 3 |
| 30/04/2022 | 30 | 37 | 45 | 3 | 4 |
| 01/05/2022 | 42 | 30 | 27 | 4 | 4 |
| 02/05/2022 | 41 | 40 | 26 | 6 | 8 |
| 03/05/2022 | 42 | 47 | 36 | 0 | 5 |
| 04/05/2022 | 36 | 39 | 44 | 2 | 4 |
| 05/05/2022 | 37 | 27 | 45 | 8 | 3 |
| 06/05/2022 | 44 | 38 | 37 | 10 | 4 |
| 07/05/2022 | 49 | 31 | 40 | 4 | 7 |
| 08/05/2022 | 33 | 26 | 20 | 1 | 1 |
| 09/05/2022 | 51 | 36 | 26 | 4 | 0 |
| 10/05/2022 | 42 | 30 | 22 | 1 | 3 |
| 11/05/2022 | 31 | 36 | 24 | 3 | 3 |
| 12/05/2022 | 41 | 42 | 24 | 5 | 2 |
| 13/05/2022 | 27 | 37 | 31 | 3 | 1 |
| 14/05/2022 | 39 | 33 | 26 | 3 | 7 |
| 15/05/2022 | 42 | 31 | 31 | 5 | 2 |
| 16/05/2022 | 47 | 40 | 35 | 1 | 4 |
| 17/05/2022 | 33 | 41 | 24 | 3 | 3 |
| 18/05/2022 | 29 | 42 | 28 | 3 | 3 |
| 19/05/2022 | 33 | 40 | 34 | 5 | 5 |
| 20/05/2022 | 34 | 36 | 22 | 1 | 0 |
| 21/05/2022 | 41 | 42 | 20 | 9 | 4 |
| 22/05/2022 | 26 | 33 | 20 | 8 | 1 |
| 23/05/2022 | 30 | 24 | 23 | 2 | 1 |
